# Comment on: “Exploring the best treatment options for BRAF-mutant metastatic colon cancer”

**DOI:** 10.1038/s41416-020-0819-5

**Published:** 2020-03-31

**Authors:** Gérard Milano, Jocelyn Gal

**Affiliations:** 1 Oncopharmacology Unit and UNS EA 7497, Centre Antoine Lacassagne, University Côte d’Azur, Nice, F-06189 France; 2 Epidemiology and Biostatistics Department, Centre Antoine Lacassagne, University Côte d’Azur, Nice, F-06189 France

**Keywords:** Molecular medicine, Oncology

An interesting and particularly welcome article by Taieb et al.^1^ describes treatment options for the management of bad-prognosis colorectal cancer (CRC) patients harbouring BRAF-mutated tumours. The current and future therapeutic strategies for this category of patients are based on well-established and complex preclinical data to which the authors paid insufficient attention.

One of the major advances in BRAF-mutated CRC is the setting of BRAFi–MEKi combinations. Of importance, although not discussed by the authors, this association of a BRAFi with a MEKi was dictated by the fact that BRAFi can induce neoplasia, most often cutaneous squamous cell carcinoma.^2^ This effect is attributed predominantly to paradoxical ERK activation (the ability of BRAFi to stimulate RAF signalling in BRAF wt condition, thus activating ERK and stimulating proliferation).^3^ Figure 1 of the paper^1^ makes no mention of this tissue-specific molecular aspect, which is important not only for mechanistic reasons, but is also of therapeutic interest since BRAFi, which evades paradoxical MAPK pathway activation, is currently in clinical development.^4^ Moreover, a definition of a paradox index has been set for BRAFi (vemurafenib, dabrafenib and encorafenib, PLX8394) as a means of quantifying a therapeutic window of high clinical efficacy (at least in BRAF-mutant melanoma cells) with minimal paradoxical ERK activation.^5^

**Fig. 1:**
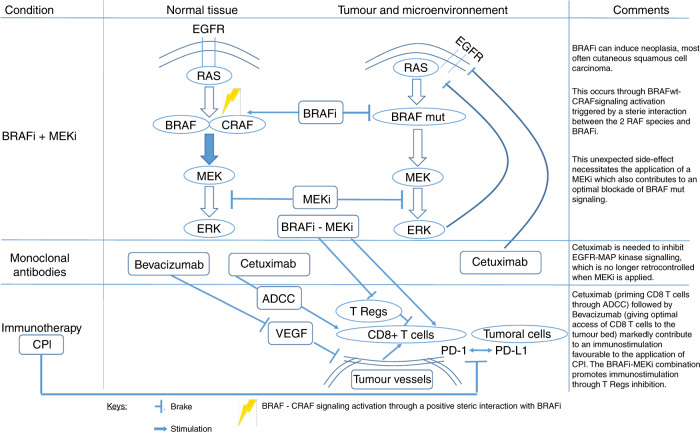
BRAF - mutated metastatic CRC: molecular bases for an optimal combination BRAFi - MEKi - cetuximab - bevacizumab - CPI.

The authors did mention another level of the underlying molecular complexity, i.e. the interruption by MEKi of negative feedback of ERK signalling at the initiation steps of MAPKinase signalling.^6^ This explains why, in the case of colorectal cancer cells and not melanoma cells (deprived of EGFR signalling), the addition of EGFRi is a necessary complement to the BRAFi–MEKi association. When advocating EGFRi in CCR, and more broadly, the use of monoclonal antibodies, the respective order of application of cetuximab versus bevacizumab is still a matter of debate. Cetuximab is an IgG1 and this characteristic confers to the drug the capacity to develop the clinically important mechanism of antibody-dependent cellular cytotoxicity (ADCC). ADCC was recently highlighted by others and us for its significant contribution to the global action mechanism of cetuximab.

Of importance is the immune modulation generated by ADCC.^7^ In brief, based on preclinical and clinical observations, cetuximab-mediated ADCC, through natural killer-cell release of INFα, results in priming of cytotoxic T cells.^7^ Recent experimental and clinical data have revealed that bevacizumab, through its interaction with VEGF, may in fact restore normal endothelial cell diapedesis. This results in favourable tissular diffusion of cytotoxic T lymphocytes instead of regulatory T cells, the diffusion of which is facilitated by the deleterious impact of VEGF on endothelial cells.^8^ Thus, under bevacizumab treatment, adequate tumoural tissue redistribution of beneficial antitumour CD8 T cells is achieved in place of detrimental regulatory T cells. This background may support recent clinical results regarding the optimal order for cetuximab (priming CD8 T cells) versus bevacizumab (allowing optimal access of CD8 T cells to the tumoural bed) in first-line metastatic CRC^9^ and strengthens combination strategies also including immunotherapy by checkpoint inhibitors in unstable microsatellite BRAF-mutated CRC patients. The fact that BRAF + MEK inhibition positively affects the tumour microenvironment and immune modulation is thus a strong argument in favour of adding immunotherapy in the context of the cetuximab–BRAFi–MEKi/bevacizumab combination with attention being paid to an optimal sequencing. The included figure recapitulates the molecular mechanisms considered above and seeks to clarify the particularly complex background-sustaining treatment options for metastatic BRAF-mutated CRC.

## Data Availability

Not applicable.
